# Small Doses Frequently Repeated

**Published:** 1884-08

**Authors:** 


### Small Doses Frequently Repeated.

In a paper read before the Cincinnati Medical Society, May 13th, Dr. John L. Davis says:

Recently the question has arisen, whether we cannot secure better results more agreeably by the administration of small doses frequently; particularly, in acute diseases. The advocates of minute, frequent doses claim for their method the following advantages over the usual plan of medication:

1. The physiological action of the remedy is more quickly secured and a less amount of medicine is required.

2. The action of evanescent medicines, such as stimulants, can be made more continuous.

3. The personal peculiarities and susceptibility of patients can be better observed and consulted.

4. The medicine is more easily administered.

These are recognized as important advantages and the only question is, can they be obtained by giving small, frequent doses?

Ringer highly indorses, under certain circumstances, such minute doses as one-third of a grain of gray powder; one-twelfth of a grain of calomel; one-sixty-fourth of a grain of tartar emetic; two drops of tincture of assafœtida; one drop of nux vomica. These doses vary in size from one-fifth to one-thirtieth of the usual dose as given by the U. S. Dispensatory, though as a rule, they are to be repeated oftener than the ordinary dose.

Fothergill says that the old fashioned drug, epsom salts, is a most excellent laxative for children in five-grain doses. And we know that the laxative effect of natural mineral waters is often due to a still smaller amount of the salt.

Bartholow and Wood both recommend minute doses of various drugs under certain conditions. For more than a year, as opportunities have offered, I have experimented with small, frequently repeated doses, and have carefully recorded the results. These I present to-night, and as far as they are of value, I ask your attention to them.

1. *Mercury*. Sept. 1, 1883, I was called to see a delicate infant which for ten weeks—since the middle of June—had suffered from diarrhœa. During the whole period it had been under the treatment of various physicians; but it appeared to be getting worse. When first seen by me, it was having twelve or fifteen thin, greenish, offensive evacuations daily, and was exceedingly debilitated and emaciated. The family had given up all hopes of its recovery, and in my opinion the prognosis was very grave. It was ordered one-eighth of a grain of calomel and three grains of sugar of milk hourly. Within a few hours the dejections became more natural and the child almost immediately began to improve. The next day its condition was so much better as to excite the wonder and gratification of the family, who were excessive in their praise of the powder. In a week the child was well. This is a typical case of long continued infantile diarrhea of unusual severity. I have notes of eight other cases of the kind, though none of them were as severe as this. In all of these cases the treatment consisted in the administration of minute doses of calomel—one-tenth to one-thirtieth of a grain, repeated every thirty minutes or one hour at first, and later at longer intervals. In every case without a single exception the treatment succeeded admirably. The same drug I have given to several adult patients in the condition characterized as 'bilious,' from one-third to one-half of a grain every hour or two; and thus given, it has invariably acted much more promptly and agreeably than the ordinary large dose of calomel or blue mass.

